# Pronounced reversible hyperammonemic encephalopathy associated with combined valproate–topiramate therapy in a 7-year-old girl

**DOI:** 10.1186/s40064-015-1057-9

**Published:** 2015-06-17

**Authors:** Sebastian Weise, Steffen Syrbe, Matthias Preuss, Astrid Bertsche, Andreas Merkenschlager, Matthias K Bernhard

**Affiliations:** Department of Women and Child Health, University Hospital for Children and Adolescents, Liebigstr. 20a, 04103 Leipzig, Germany; Department of Neurosurgery, Pediatric Neurosurgery, University Hospital Leipzig, Liebigstrasse 20, 04103 Leipzig, Germany

**Keywords:** Topiramate, Valproate, Encephalopathy, Glutamine, Hyperammonemia

## Abstract

Valproate is one of the most frequently used anticonvulsive drugs in children and adults. Valproate is a generally well tolerated medication. However, encephalopathy with or without hyperammonemia is one of its rare adverse events. We present a 7-year-old girl who suffered from epilepsy with generalized tonic–clonic seizures and absence epilepsy. She was initially treated with topiramate. Methylprednisolone pulse therapy and long-term therapy with valproate were initiated due to an increase of seizure frequency. At day 5 of therapy, a further increase of seizure frequency was observed followed by lethargy and somnolence. Liver enzymes remained within normal range, but ammonia serum levels increased to a maximum of 544 mmol/l. Discontinuing valproate and starting potassium-benzoate and sodium-phenylbutyrate improved the clinical condition and ammonia serum levels. Haemodialysis was not required. Cranial magnetic resonance imaging ruled out brain edema. The patient was further on successfully treated with a combination of both, topiramate and levetiracetam. Seizures did not recur and development was normal until now (3 years later). To the best of our knowledge, we observed the highest ammonia serum levels ever reported in valproate-induced hyperammonemia with a complete remission of the subsequent encephalopathy. Topiramate might increase the risk of valproate-induced encephalopathy by carbonic anhydrase inhibition.

## Background

Valproate is one of the most frequently used anticonvulsive drugs in pediatric patients. It is mainly used for epilepsy with generalized seizures and absence epilepsy. Usually, valproate is well tolerated. Several adverse events have been associated with to VPA treatment, including sleepiness and weight gain and less frequently thrombocytopenia, hepatopathy or encephalopathy. Hyperammonemia with or without evidence of hepatic toxicity is an important but very rare idiosyncratic adverse event of valproate. Valproate-induced hyperammonemic encephalopathy that is a potentially acute life-threatening condition if it is not treated appropriately.

We report a 7-year-old girl who suffered from a reversible valproate-induced hyperammonemic encephalopathy during combination therapy of VPA with topiramate. The consent to publish this case report was obtained by the mother of the child.

## Case report

A healthy and normally developed 7-year-old girl experienced a first generalized tonic–clonic seizure, lasting 2 min. Additionally absence seizures were recorded and EEG displayed typical 3 Hz spike-wave complexes. Cranial magnetic resonance imaging (MRI) displayed no pathological findings. Topiramate was titrated slowly over 8 days up to 100 mg (2 × 50 mg) per day (4.4 mg/kg body weight/day) resulting in cessation of absence seizures.

On day 11 of treatment she experienced 20 min lasting generalized seizure relapse that was only terminated by intravenous application of diazepam and midazolam. EEG recording at this time was consistent with absence status epilepticus. Add-on therapy with valproate 900 mg (2 × 450) per day (39 mg/kg body weight/day) was started. An additional intravenous methylprednisolone pulse therapy was given for 3 days because of recurring generalized tonic–clonic seizures and absences. At the end of methylprednisolone treatment (day 21) blood count, ALT (alanine transaminase), AST (aspartate transaminase), GGT (gamma-glutamyl transferase) showed normal values, ammonia 41 mmol/l (reference 15–55 mmol/l), valproate 96 mg/l (reference 50–100 mmol/l).

One day after cessation of methylprednisolone pulse therapy (day 22) seizure frequency increased again. The patient appeared progressively apathetic, somnolent and finally comatose. Breathing was not impaired. Laboratory findings displayed the following: ALT 0.13 µkat/l, AST 0.26 µkat/l, ChE (cholinesterase) 121 µkat/l, GLDH (glutamate dehydrogenase) <0.02 µkat/l, NSE (neuron-specific enolase) 12.6 µg/l, valproate 104 mg/l, ammonia 544 mmol/l. Valproate was immediately terminated. The seizures were halted by infusion of midazolam and intravenous levetiracetam (40 mg/kg body weight/day), as well as intravenous sodium benzoate, sodium phenylbutyrate and high-dose glucose.

For haemodialysis the patient would have been transferred to a nearby dialysis center. The medical team decided against the transfer because of the critical condition of the girl on day 22. On day 23 the ammonia serum level was decreased to 245 mmol/l. A haemodialysis was not seen to be justified anymore on this therapeutical progress.

On day 23 (the patient was comatose) EEG revealed generalized delta slowing and single generalized epileptic discharges being consisted with metabolic encephalopathy. Seizures ceased quickly and the patient had a normal level of consciousness again (day 25).

On day 30 EEG showed no epileptiform discharges and ammonia serum level had returned to normal range (53 mmol/l). Subsequent ammonia serum levels were measured between 20 and 30 mmol/l. ALT, AST and GGT stayed within normal ranges, as well as amino acid levels in plasma, acylcarnitines in serum and organic acids in urine. The patient continued therapy with topiramate (100 mg per day) and levetiracetam (1,000 mg per day). EEG as well as cranial MRI remained normal. Seizures did not recur and development was normal until now (3 years later).

## Discussion

We present the case of a 7-year-old girl who displayed a distinct hyperammonemic encephalopathy. Liver enzymes were not elevated. Likely cause of the hyperammonemia was the therapy consisting of both, topiramate and valproate. Ammonia levels reached from normal serum levels within a day a maximum 544 mol/l.

Apart from pancreatitis, coagulopathy and bone marrow suppression, hepatic toxicity and encephalopathy are rare but long known adverse events of valproate therapy independent of patient’s age (Gerstner et al. 2008). Symptoms of valproate-induced hyperammonemic encephalopathy are impaired level of consciousness, focal neurological symptoms and increased seizure frequency (Eubanks et al. 2008; Hamer et al. 2000; McCall and Bourgeois 2004; Verrotti et al. 2002). Mental retardation, carnitine deficiency and urea cycle defects increase its risk (Chopra et al. 2012). Patients develop both transaminase elevation and isolated hyperammonemia without transaminase elevation with valproate-induced encephalopathy (Amanat et al. 2013; Verrotti et al. 2002). Until today, the highest ammonia value of 411 mmol/l has been reported in a 47-year-old man who came up with a completely reversible valproate-induced encephalopathy. In contrast to our report, this patient’s MRI disclosed a significant brain edema followed by cerebral atrophy (Hantson et al. 2005).

The present case reports the youngest patient ever reported with a valproate-induced encephalopathy. Additionally it seems to be the highest ammonia serum level ever published so far without leading to persistent cerebral damage. Fortunately, we did not observe any long-term pathology in the patient’s MRI which might be due to immediate therapy of hyperammonemia which resulted in normal ammonia serum levels within 8 days. In contrast to other cases, a mild clinical course was encountered despite of extraordinary high ammonia levels. It may indicate a higher tolerance of ammonia in a child’s brain compared to adults. Methylprednisolone pulse therapy is more and more considered to be an efficient and mainly safe therapy in child epilepsy that is difficult to treat (Bast et al. 2014). Moreover, the previous methylprednisolone therapy might have prevented brain edema and neurological sequelae in the presented patient.

Carnitine administration has been shown to decrease hyperammonemia and hepatic toxicity in patients with valproate-induced encephalopathy (Mock and Schwetschenau 2012). However; a correlation between ammonia correlations and the clinical condition has not been proved (Lheureux and Hantson 2009). This can be especially helpful in mild cases when discontinuation of valproate therapy is not a useful option as in psychotic disorder patients (Sonik et al. 2013). Some authors recommend prophylactic carnitine supplementation in patients with risk factors of carnitine deficiency such as intake of several antiepileptic drugs, young age, intellectual disability or parental feeding (Fukada et al. 2014).

There are reports about topiramate increasing the risk for valproate-induced encephalopathy (Gomez-Ibanez et al. 2011; Hamer et al. 2000). The youngest patient reported was a 15-year-old boy carrying an inverted duplication of chromosome 15. He developed a hyperammonemic encephalopathy 2 weeks after addition of topiramate to his anticonvulsive therapy with valproate. In this case, ammonia serum levels did only double (Cheung et al. 2005). A recent study from South Korea analyzed 8,372 patients having been treated with valproate, 1,236 in combination with topiramate. 11 patients suffered valproate-induced encephalopathy. Seven of these patients had been treated with the combination of valproate and topiramate. The authors concluded a tenfold higher risk of valproate-induced encephalopathy under combination of valproate and topiramate than under valproate monotherapy (Noh et al. 2013).

Hyperammonemia is supposed to inhibit glutamate uptake into astrocytes. Subsequently, glutamate synthesis is increased and intracellular osmolarity rises. Thereby, cerebral edema is promoted (Verrotti et al. 2002). Elevated glutamate levels in serum and cerebrospinal fluid observed in patients with valproate-induced encephalopathy; support this notion (Vossler et al. 2002). Additionally the astrocyte function is impaired by the downregulation of astrocytic glutamate receptors due to elevated extracellular glutamate. This in turn leads to decreased glutathione synthesis which causes more oxidative stress for neurons and glial cells (Verrotti et al. 2002). Topiramate might enhance this mechanism by two ways. It inhibits carbonic anhydrase and thus, elevates ammonia serum levels by decreasing urea synthesis (Dodgson et al. 2000). Additionally, topiramate interacts with the cerebral glutamine synthetase possibly increasing an already existing hyperammonemia (Hamer et al. 2000).

## Conclusion

In summary, in patients with increasing seizure frequency and valproate therapy, physicians should be aware of a beginning hyperammonemic encephalopathy, even when the patient’s consciousness is mildly impaired only. In suspicious situations transaminases as well as ammonia levels should be determined. The present case confirms that assumption that topiramate is has the potential to increase the risk of valproate-induced encephalopathy.
